# S-SCAM inhibits Axin-dependent synaptic function of GSK3β in a sex-dependent manner

**DOI:** 10.1038/s41598-022-08220-1

**Published:** 2022-03-08

**Authors:** Gillian Kearney, David Grau, Damaris Nieves Torres, Seung Min Shin, Sang H. Lee

**Affiliations:** 1 Department of Pharmacology and Toxicology, Medical College of Wisconsin, 8701 Watertown Plank Road, Milwaukee, WI USA; 2 Neuroscience Research Center, Medical College of Wisconsin, 8701 Watertown Plank Road, Milwaukee, WI USA

**Keywords:** Diseases of the nervous system, Molecular neuroscience

## Abstract

*S-SCAM/MAGI-2* gene duplication is associated with schizophrenia (SCZ). S-SCAM overexpression in the forebrain induces SCZ-like phenotypes in a transgenic (Tg) mouse model. Interestingly, S-SCAM Tg mice show male-specific impairments in synaptic plasticity and working memory. However, mechanisms underlying the sex-specific deficits remain unknown. Here we report that S-SCAM Tg mice have male-specific deficits in synaptic GSK3β functions, as shown by reduced synaptic protein levels and increased inhibitory phosphorylation of GSK3β. This GSK3β hyper-phosphorylation was associated with increased CaMKII activities. Notably, synaptic levels of Axin1, to which GSK3β binds in competition with S-SCAM, were also reduced in male S-SCAM Tg mice. We demonstrated that Axin-binding is required for the S-SCAM overexpression-induced synaptic GSK3β reduction. Axin stabilization using XAV939 rescued the GSK3β deficits and restored the temporal activation of GSK3β during long-term depression in S-SCAM overexpressing neurons. Interestingly, synaptic Axin2 levels were increased in female S-SCAM Tg mice. Female sex hormone 17β-estradiol increased Axin2 expression and increased synaptic GSK3β levels in S-SCAM overexpressing neurons. These results reveal the role of S-SCAM in controlling Axin-dependent synaptic localization of GSK3β. Moreover, our studies point out the pathological relevance of GSK3β hypofunction found in humans and contribute to understanding the molecular underpinnings of sex differences in SCZ.

## Introduction

Synaptic scaffolding molecule (S-SCAM; also called membrane-associated guanylate kinase inverted 2 [MAGI-2]) is one of the major postsynaptic scaffolding molecules present at synapses^1–4^. S-SCAM interacts with many postsynaptic proteins including Axin, transmembrane AMPA receptor regulatory protein (TARP), and GKAP/SAPAP^5–7^. S-SCAM dictates the strength of excitatory synaptic transmission by controlling the amounts of AMPA receptors (AMPAR) at synapses through its TARP interaction^1,5,8^. Importantly, mutations in the *S-SCAM* gene including duplication are found in patients with SCZ^9–12^. Mimicking the duplication conditions, S-SCAM overexpression in cultured hippocampal neurons enhanced excitatory synaptic transmission^1^, impaired GABAergic synaptic transmission^13^, and impaired synaptic plasticity^1,14^. Therefore, it was expected that elevated S-SCAM levels lead to deficits in cognitive function and behavioral abnormalities. Indeed, S-SCAM Tg mice that overexpress S-SCAM under the forebrain-specific Ca^2+^-dependent protein kinase II a (CaMKIIα) promoter showed a remarkably wide array of SCZ-related behavioral endophenotypes modeling all three domains (positive, negative, and cognitive) of SCZ symptoms^14^. Notably, the S-SCAM Tg mice showed interesting sex differences. Male Tg mice showed more severe SCZ-related endophenotypes than female Tg mice. Moreover, only male S-SCAM Tg mice showed deficits in long-term potentiation (LTP). These findings are, in general, consistent with the sex differences observed in SCZ^15^. However, the molecular and physiological bases of the sex differences in SCZ are still poorly understood.

Precise temporal control of GSK3 activity is crucial for the induction of LTP and long term depression (LTD)^7,16–18^. GSK3 is encoded by two genes producing GSK3α and GSK3β isoforms that have similar catalytic activity. GSK3β is the major isoform expressed in the brain^19^ and is present in dendritic spines in addition to the soma and dendrites of neurons^17^. GSK3 is constitutively active but the phosphorylation of its Ser residue (pS9 for GSK3β; pS21 for GSK3α) inactivates the kinase^19,20^. During LTD, GSK3β is activated by PP1-mediated de-phosphorylation of pS9. Activated GSK3β phosphorylates Thr19 of PSD-95 to promote the immobilization of PSD-95 at the synapse, which is a prerequisite for AMPA receptor internalization during LTD^21^. On the other hand, the inactivation of GSK3β is required for LTP formation^16,17^.

Axin is a critical scaffolding protein for forming the GSK3β‒β-catenin destruction complex in the canonical Wnt signaling^22^. Axin is encoded by two genes producing highly homologous proteins, Axin1 and Axin2 (also known as Axil/conductin)^23^. Axin is enriched at dendritic spines^7,24^ and is important for the axonal localization of GSK3β in developing neurons^25,26^. S-SCAM binds to the middle region of Axin1 protein (containing GSK3β-interacting domain [GID]) through its guanylate kinase (GK) domain^7^. The GIDs of Axin1 and Axin2 are highly conserved (~ 70% similarity at the amino acid sequence level). Since S-SCAM and GSK3β bind the same region in Axin, S-SCAM was shown to compete with GSK3β for Axin-binding^7,26^. This competitive binding inhibits β-catenin phosphorylation by GSK3β in vitro^7^. Therefore, it is likely that GSK3β interaction with Axin is hampered in S-SCAM Tg mice, which may disrupt the proper synaptic localization of GSK3β in neurons. However, these possibilities have not been addressed.

As a first step to understand the molecular bases of the sex differences exhibited in S-SCAM Tg mice, we investigated whether aberrant GSK3β signaling is associated with these phenotypes, as well as the potential role of Axin and female sex hormone in the process. These studies reveal the role of Axin in maintaining proper GSK3β signaling at synapses and provide mechanisms underlying the sex differences found in S-SCAM Tg mice.

## Results

### Synapse-specific reduction of GSK3β protein levels and its activity in male S-SCAM Tg mice

**Figure 1:**
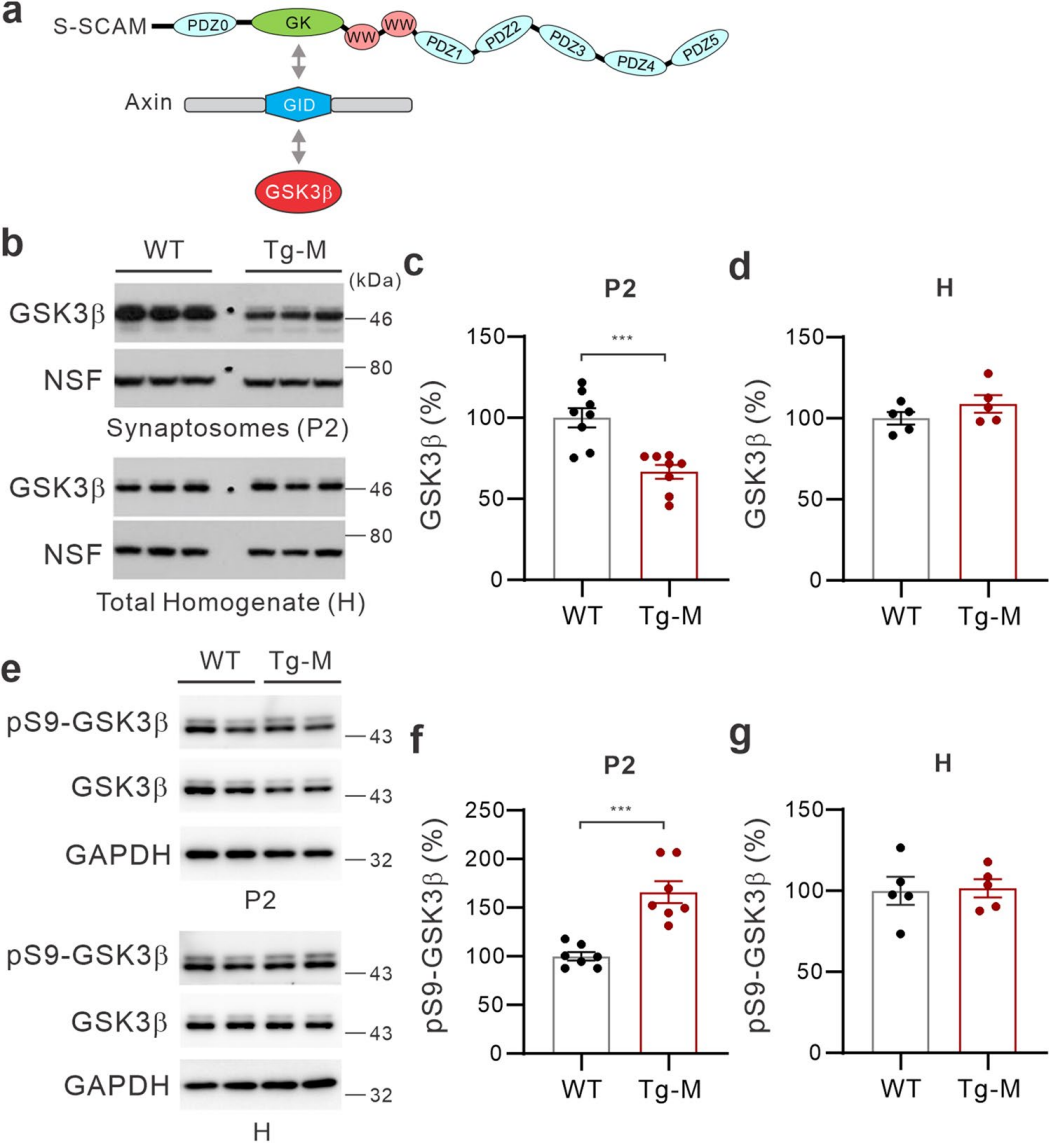
Reduced synaptic GSK3β protein levels and hyper-phosphorylation of GSK3β in the forebrain tissues of 3-month-old male S-SCAM Tg mice. (**a**) Schematic diagram showing the competitive binding (↔) of S-SCAM with GSK3β for the GID domain of Axin. (**b**–**d**) GSK3β protein levels in the synaptosomal fraction (P2) and total homogenate (H) of male S-SCAM Tg mice. Representative results (**b**) and quantification (**c**, **d**). *n* = 5–8 mice per group. *N*-ethylmaleimide-sensitive factor (NSF) was used as loading control. (**e**–**g**) pS9-GSK3β protein levels in the P2 and H fractions. Representative results (**e**) and quantification of relative pS9-GSK3β levels normalized to GSK3β protein levels (**f**, **g**). *n* = 5–7 mice per group. ***p < 0.001, unpaired *t*-test with Welch’s correction. n.s., not significant.

To study the role of S-SCAM in GSK3β-mediated signaling at synapses (Fig. 1a), we first examined GSK3β protein levels in synaptosomal fractions (P2; biochemical correlates of synapses^27^) of the forebrain tissues obtained from male Tg mice (Tg-M; 3 ± 0.5-month-old). GSK3β protein levels in P2 fractions were greatly reduced (66.7% ± 4.3% of WT; Fig. 1b, c). On the other hand, GSK3β levels in total homogenate fraction (H) of male Tg mice show no difference from male WT mice (Fig. 1b, d), suggesting that the reduced synaptic GSK3β protein levels are unlikely due to the decreased expression of GSK3β. This synaptic GSK3β reduction is accompanied by increased synaptosomal phospho-Ser9-GSK3β (pS9-GSK3β) levels (normalized to GSK3β levels; 165.9% ± 11.4% of WT; Fig. 1e, f). In contrast, pS9-GSK3β levels in total homogenate (H) of male Tg mcie showed no difference from male WT mice (Fig. 1e, g). These results suggest that S-SCAM overexpression inhibits synaptic targeting of GSK3β and impairs synaptic GSKβ activity in the brain of male S-SCAM Tg mice.

### Elevated CaMKII activity in the brains of male S-SCAM Tg mice

**Figure 2:**
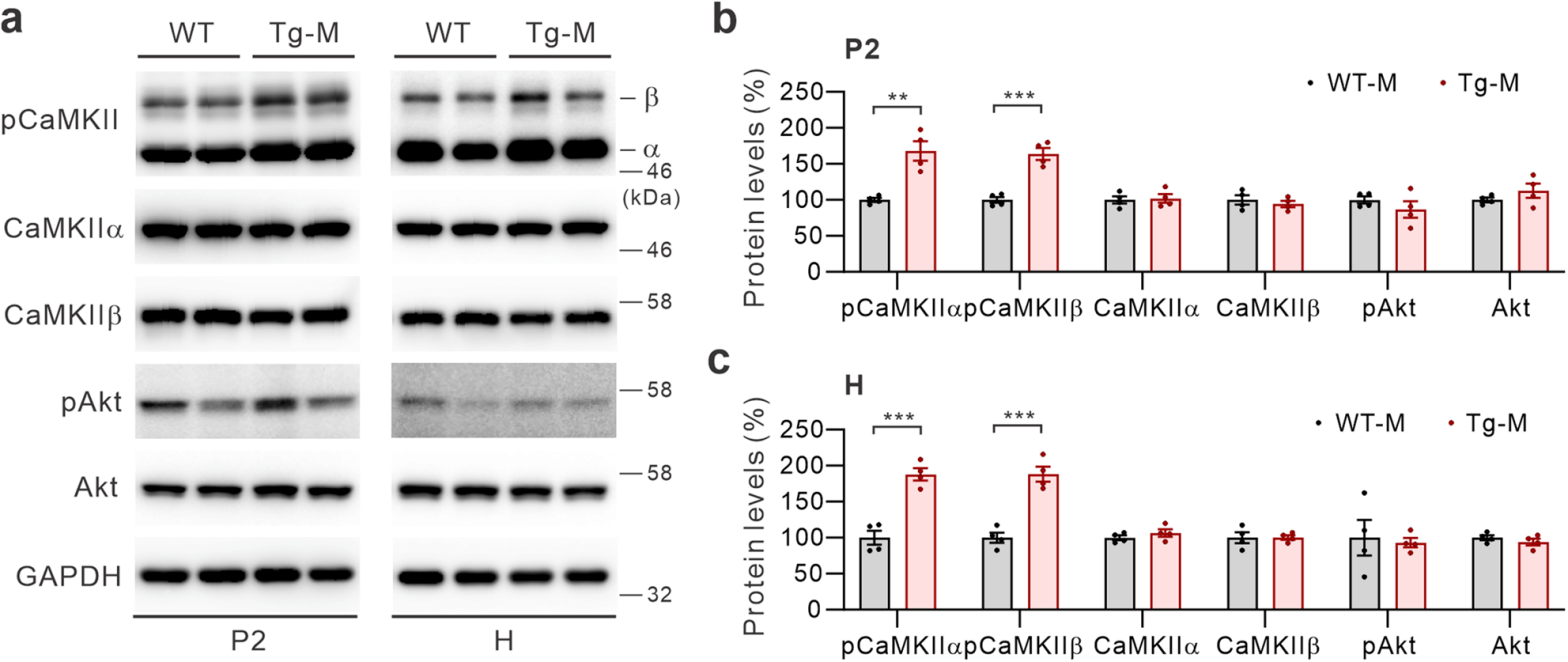
Enhanced CaMKII activity, not Akt, is associated with the hyper-phosphorylation of GSK3β in male S-SCAM Tg mice. (**a**) Representative immunoblots showing pCaMKIIs, CaMKIIα, CaMKIIβ, pAkt, and Akt protein levels in the P2 (left) and H fractions (right) of male S-SCAM Tg mice. (**b**–**c**) Quantification of P2 (**b**) and H data (**c**). *n* = 4 mice per group. ***p < 0.001, **p < 0.01, unpaired *t*-test.

To investigate the mechanisms associated with the enhanced inhibitory phosphorylation of synaptic GSK3β in the brains of male S-SCAM Tg mice, we examined the activity of two known upstream protein kinases of GSK3β, CaMKII and Akt^19,28^. Activation of these kinases were assessed by specific antibodies recognizing phosphorylated (and thus activated) forms of CaMKIIa and CaMKIIb (pThr286 and pThr287 for CaMKIIa and CaMKIIb, respectively) and Akt (pSer473). We found that total pCaMKII levels were significantly increased in both P2 and H fractions of male Tg mice (168% ± 13.6% and 188% ± 8.6% of WT, respectively; Fig. 2a–c). On the other hand, both pAkt and Akt protein levels were not significantly changed in either P2 or H fractions (Fig. 2a–c). These results suggest that elevation of inhibitory phosphorylation of GSK3β is most likely caused by increased CaMKII activity in male S-SCAM Tg mice. These data are consistent with elevated glutamatergic activity at the synapse found in S-SCAM Tg mice^14^.

### Reduced synaptic GSK3β protein levels in male S-SCAM Tg mice is associated with decreased synaptic Axin1 levels

**Figure 3:**
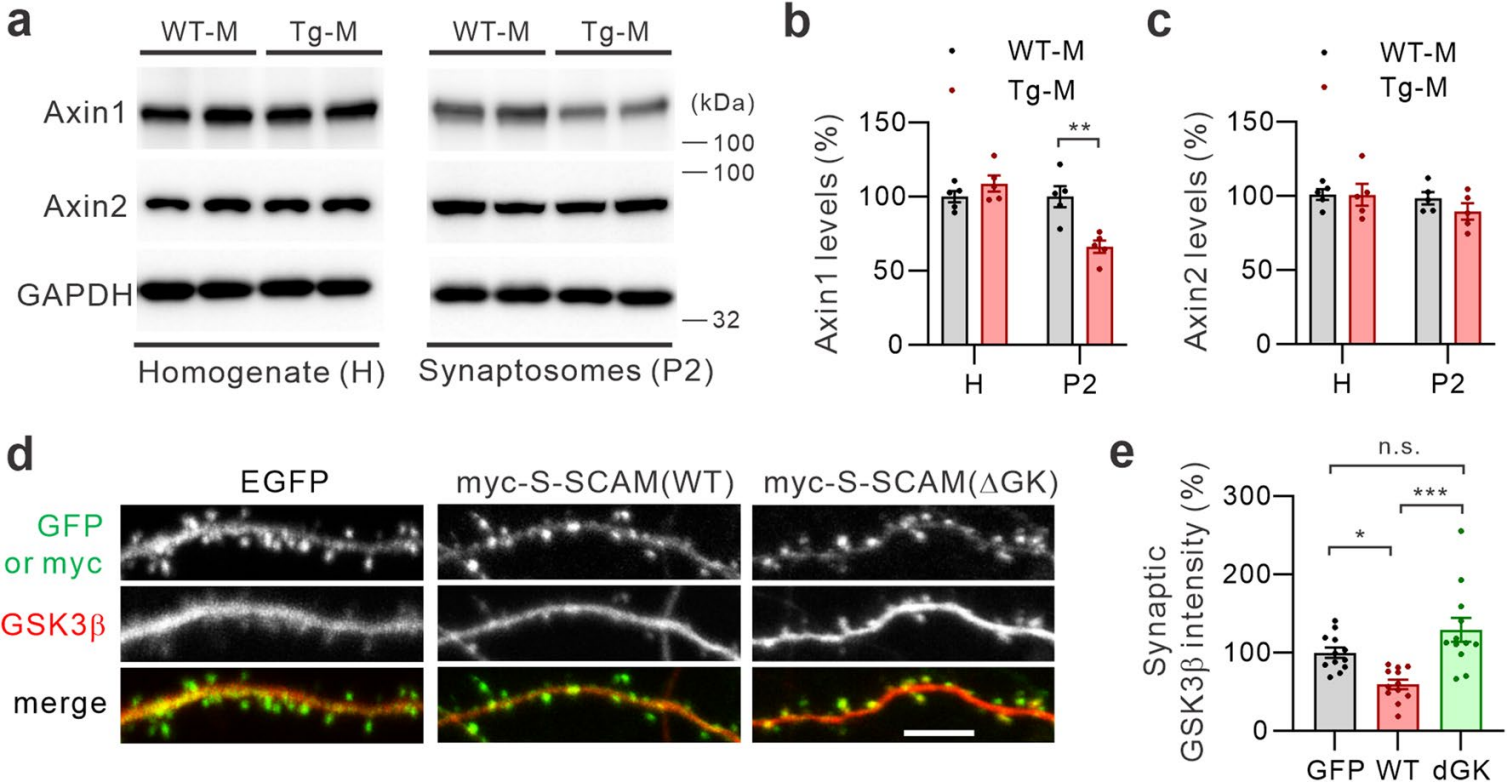
Altered synaptic Axin1 levels are responsible for the reduction of synaptic GSK3β. (**a**–**c**) Reduced Axin1 levels in the P2 fraction of male S-SCAM Tg mice. Representative immunoblots (**a**), quantification of Axin1 (**b**) and Axin2 protein levels (**c**). *n* = 5 mice per group. **p < 0.01, unpaired *t*-test with Welch’s correction. (**d**, **e**) Removal of the GK domain in S-SCAM prevents the loss of synaptic GSK3β in cultured hippocampal neurons. Representative images (**d**) and quantification of synaptic GSK3β immunofluorescent intensity (**e**). *n* = 12 neurons per group. One-way ANOVA, F _(2,33)_ = 11.62, *p* < 0.001. Tukey’s multiple comparison test: ***p < 0.001, *p < 0.05. Scale bars represent 5 mm.

Axin1 is one of the S-SCAM-interacting proteins and is known to be involved in the recruitment of GSK3β to the plasma membrane^29^. To investigate the potential role of Axin in the altered synaptic localization of GSK3β in male S-SCAM Tg mice, we first explored the possibility that Axin protein levels are altered in the S-SCAM Tg mice. We examined both Axin1 and Axin2 proteins, since they can be functionally interchangeable^23^. Total protein levels of both Axin1 and Axin2 in the forebrain tissues of male Tg mice were indistinguishable from male WT mice (Fig. 3a, b). In contrast, Axin1 protein levels in the P2 fraction were significantly reduced in male S-SCAM Tg mice (66% ± 4.2% of WT; Fig. 3a, b). On the other hand, there was no such difference in Axin2 protein levels in the P2 fraction (Fig. 3a, c). Therefore, male S-SCAM Tg mice have Axin1 deficits in synapses of the forebrain.

S-SCAM and GSK3β competitively bind the same region in Axin^7^. Therefore, S-SCAM overexpression may hamper the Axin-mediated synaptic targeting of GSK3β. To address this possibility, we took advantage of the fact that Axin binds to the GK domain of S-SCAM^7^. Therefore, a S-SCAM mutant lacking the GK domain (DGK)^13^ should not interfere with the Axin- GSK3β interaction. The DGK mutant is indistinguishable from WT S-SCAM in synaptic targeting, as well as its ability to increase spine sizes and synaptic AMPA receptor levels^13^. Consistent with our hypothesis, while the overexpression of WT S-SCAM greatly reduced synaptic GSK3β levels (59.7% ± 6% of GFP control; Fig. 3d, e), the overexpression of DGK mutant did not hamper the synaptic targeting of GSK3β (127% ± 15.2%; Fig. 3d, e). These results strongly suggest S-SCAM overexpression inhibits Axin-mediated synaptic targeting of GSK3β.

### Stabilization of Axin1 restores synaptic targeting and temporal regulation of GSK3β activity during LTD

**Figure 4:**
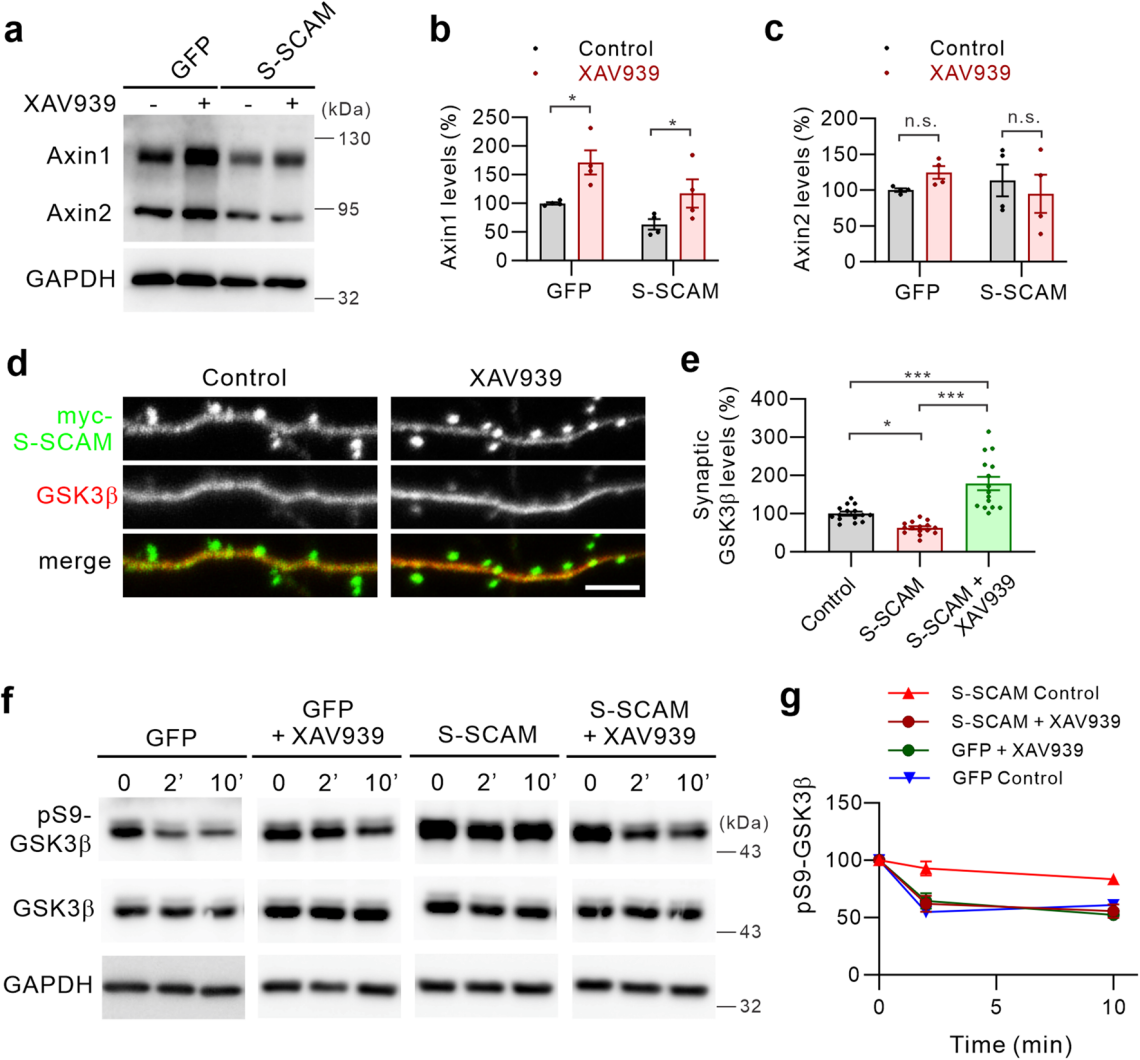
Axin stabilization restores synaptic GSK3β levels and its temporal regulation during Chem-LTD. (**a**–**c**) Effect of XAV939 (5 mM) on the total protein levels of Axin1 and Axin2 in GFP- or S-SCAM-overexpressing cultured hippocampal neurons. Representative immunoblots (**a**), and quantification of the data for Axin1 (**b**) and Axin2 (**c**). *n* = 4 per group. *p < 0.05, unpaired *t*-test. (**d**, **e**) Effect of XAV939 on synaptic GSK3β levels in myc-S-SCAM overexpressing neurons. Representative images (**d**), and quantification of the data (**e**). *n* = 15 per group. One-way ANOVA, F _(2,42)_ = 29.91, *p* < 0.001. Tukey’s multiple comparison test: ***p < 0.0001, *p < 0.05. (**f**–**g**) Effect of XAV939 on the GSK3β activation during Chem-LTD. Representative immunoblots (**f**) and quantification of the data (**g**). *n* = 3 per group. *p < 0.05, unpaired *t*-test with Welch’s correction.

To further study the role of Axin1 deficits in the impairment of GSK3β function at synapses, we thought to rescue the deficits using pharmacological approaches. A small molecule tankyrase inhibitor XAV939 stabilizes Axin by preventing ADP-ribosylation and subsequent degradation^30,31^. Incubation of cultured hippocampal neurons transfected with GFP-expressing Sindbis virus with XAV939 (5 mM) for 24 h greatly increased the total amount of Axin1 (171.3% ± 21% of GFP control; Fig. 4a, b).Total Axin1 levels in cultured hippocampal neurons infected with Sindbis virus overexpressingS-SCAM (Supplementary Fig. 1) were reduced (63.1% ± 9% of GFP control), although the reduction was not statistically significant (p = 0.4462). Nonetheless, XAV939 significantly increased the amount of total Axin1 in S-SCAM overexpressing cultured hippocampal neurons (186% ± 39% of S-SCAM control; 117.4% ± 25% of GFP control; Fig. 4a, b). Unexpectedly, we did not see significant changes in the amount of total Axin2 after XAV939 treatment in both GFP and S-SCAM overexpressing neurons (Fig. 4a, c).

Having verified the restoration of Axin1 levels in S-SCAM overexpressing neurons, we next examined the effect of XAV939 on synaptic GSK3β levels in S-SCAM overexpressing neurons. Incubation of hippocampal neurons with XAV939 greatly increased synaptic GSK3β staining intensity (178% ± 19% of GFP control; cf. 62% ± 5% for S-SCAM control; Fig. 4d, e), indicating increased synaptic GSK3β protein levels. In contrast, XAV939 did not change synaptic GSK3β levels in GFP-transfected neurons (Supplemental Fig. 2).

We showed previously that the overexpression of S-SCAM blocks LTD formation in both cultured hippocampal neurons and hippocampal slices^1^. To evaluate the restoration effect of Axin1 and GSK3β protein levels at synapses on synaptic plasticity, we examined the temporal changes of GSK3β activity during LTD which is critical for LTD formation^19^. We used a chemically (NMDA)-induced LTD (chem-LTD) protocol, which mimics the low frequency stimulation-induced LTD mechanistically^21,32^. We first examined whether XAV939 by itself had an effect on GSK3β activation during LTD. XAV939 alone did not affect NMDA-induced activation of GSK3β in GFP overexpressing neurons, as shown by the rapid reduction in the inhibitory phosphorylation of S9 (Fig. 4f, g). This is similar in temporal profile to GFP control and to previous reports obtained from naïve hippocampal neurons^17,21,33^. Having confirmed that XAV939 does not affect the rapid activation of GSK3β upon NMDA treatment, we next examined the changes of GSK3β activity during chem-LTD in S-SCAM overexpressing neurons. Surprisingly, pS9-GSK3β levels were not significantly changed upon NMDA treatment in S-SCAM overexpressing neurons, indicating dysregulated GSK3β activity. Remarkably, XAV939 restored the temporal activation of GSK3β in S-SCAM overexpressing neurons, which is indistinguishable from XAV939-treated GFP control neurons (Fig. 4f, g). These results collectively suggest that S-SCAM overexpression hampers the temporal regulation of GSK3β activity and blocks LTD formation by inducing Axin1 deficits at synapses.

### 17β-Estradiol (E2) increases Axin2 expression and preserves synaptic GSK3β protein levels

**Figure 5:**
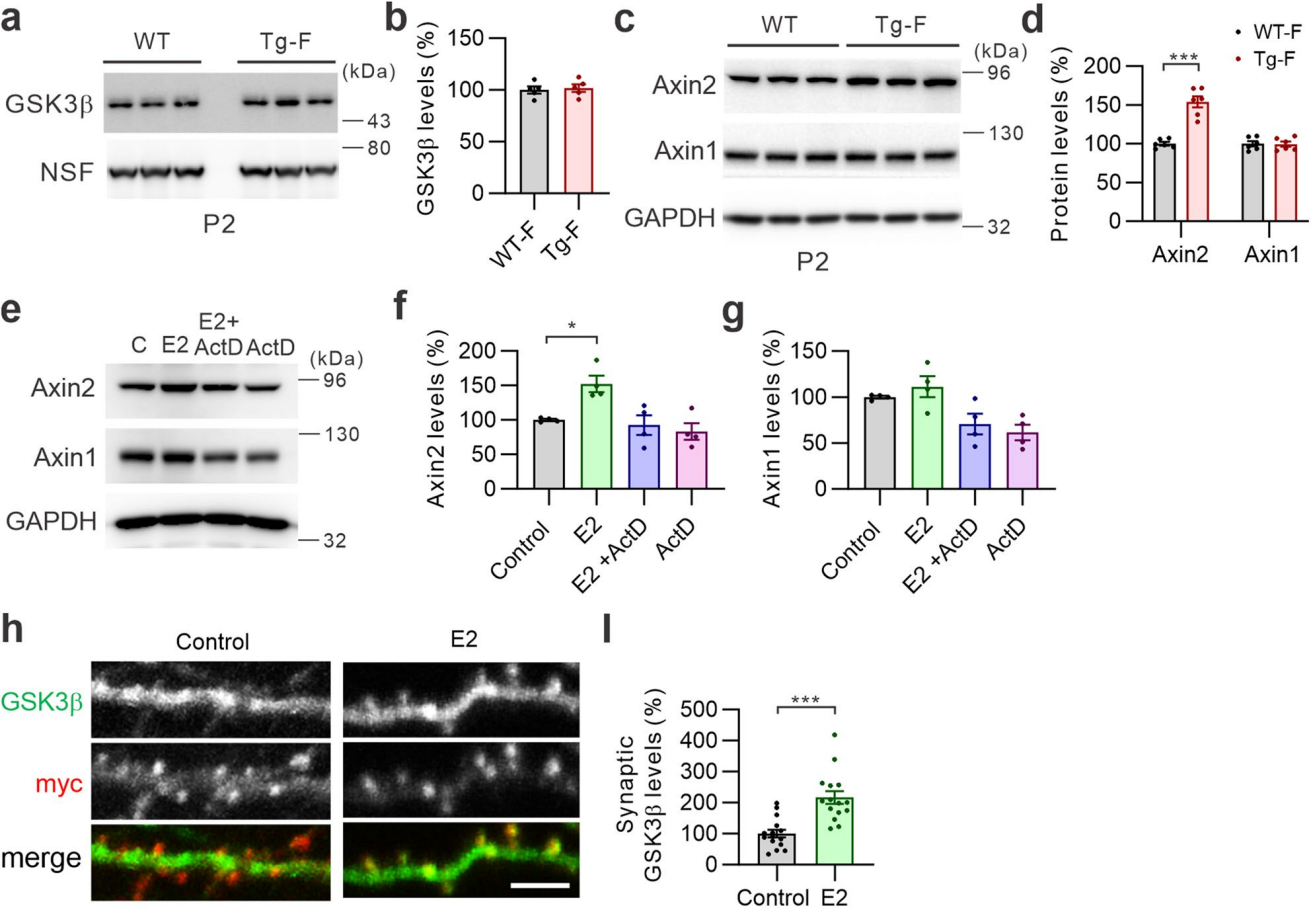
Sex-specific reduction of synaptic GSK3β is mediated by estradiol-mediated increase of Axin2. (**a**, **b**) Female S-SCAM Tg mice have normal synaptosomal GSK3β protein levels. Representative images (**a**) and quantification of GSK3β levels in the P2 fraction (**b**). *n* = 5 per group. (**c**, **d**) Increased Axin2 protein levels in the P2 fraction of female S-SCAM Tg mice. Representative images (**c**) and quantification of Axin1 and Axin2 levels in the P2 fraction (**d**). *n* = 6 per group. ***p < 0.0001, unpaired *t*-test with Welch’s correction. (**e**–**g**) E2 (10 nM) increases Axin2 protein levels in cultured hippocampal neurons. Representative images (**e**) and quantification of Axin1 (**f**) and Axin2 levels (**g**). *n* = 4 per group. One-way ANOVA, F _(3,12)_ = 7.856, *p* < 0.01. Tukey’s multiple comparison test: *p < 0.05. (**h**, **i**) Effect of E2 on synaptic GSK3β levels in myc-S-SCAM overexpressing neurons. Representative images (**h**) and quantification of the data (**i**). *n* = 15 per group. ***p < 0.001, unpaired *t*-test with Welch’s correction.

To understand the male-specific deficits in synaptic plasticity in S-SCAM Tg mice, we examined whether female S-SCAM Tg mice have alterations in GSK3β and Axin protein levels in the P2 fractions. Unlike male Tg mice, female Tg mice do not show alterations in synaptic GSK3β (Fig. 5a, b) and Axin1 protein levels (Fig. 5c, d). Surprisingly, synaptic Axin2 protein levels were significantly increased in the female Tg mice (154% ± 7% of WT; Fig. 5c, d). These results suggest that female-specific alteration in Axin2 may have a protective effect in preserving synaptic GSK3β function.

Axin2, while functionally similar to Axin1, shows distinguished expression patterns from Axin1^23^. For example, it was shown that Axin2 expression is inducible by Wnt signaling^34,35^. To investigate the effect of female sex hormone E2 on Axin2, we treated cultured hippocampal neurons with E2 (100 nM) and examined its effect on total Axin protein levels. As shown in Fig. 5e, E2 significantly increased the amount of Axin2 protein (149% ± 13% of control; Fig. 5f), while Axin1 protein levels were not affected much (Fig. 5g). This E2-induced Axin2 protein increase was blocked by the transcription inhibitor actinomycin D (ActD, 2 mM; Fig. 5e, f), suggesting that E2 induces *Axin2* expression. Notably, Axin1 protein levels were decreased by ActD (61.5% ± 13% of control; Fig. 5g), although the data did not reach statistical significance (*p* = 0.051).

Since Axin stabilization increases synaptic GSK3β protein levels in S-SCAM-overexpressing neurons (Fig. 4e), we next examined the effect of E2 on synaptic GSK3β protein levels in S-SCAM overexpressing neurons. Compared to control neurons, E2-treated neurons showed a great increase in synaptic GSK3β staining intensities (217% ± 21%; Fig. 5h, i). E2 did not have significant effect on synaptic GSK3β levels in GFP-transfected neurons (Supplemental Fig. 2). These results suggest that E2 preserves synaptic GSK3β function by increasing Axin2 expression in female Tg mice.

### S-SCAM overexpression did not change the amount of β-catenin

**Figure 6:**
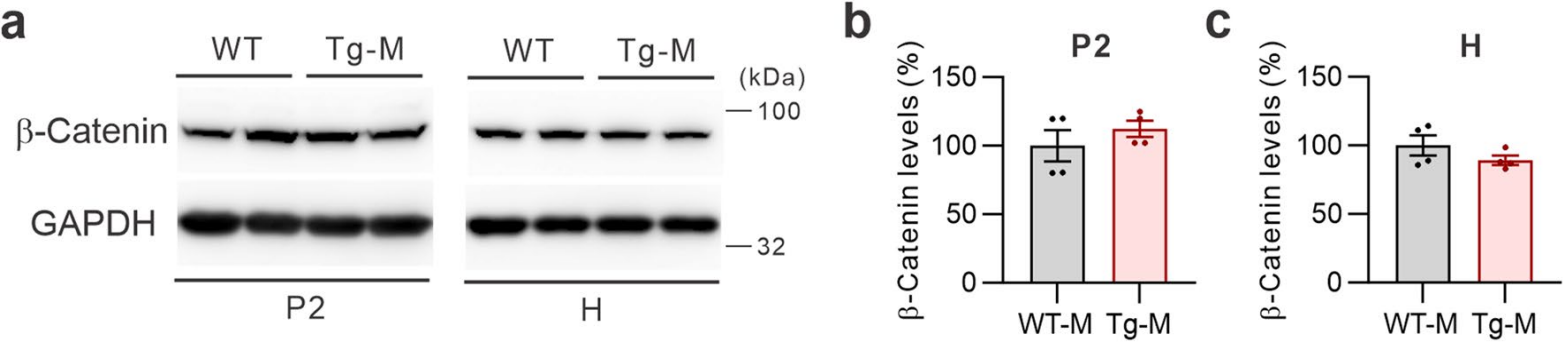
Total and synaptic β-catenin protein levels in S-SCAM male Tg mice. (**a**) Representative immunoblots showing β-catenin and GAPDH protein levels in the P2 (*left*) and H fractions (*right*) of male S-SCAM Tg mice. (**b**, **c**) Quantification of P2 (**b**) and H data (**c**). *n* = 4 mice per group.

Axin is considered as a key limiting factor for canonical Wnt signaling since it controls β-catenin levels by promoting the assembly of β-catenin destruction complex. In neurons, however, β-catenin also plays a structural function at synapses, independent of its role in the transcriptional regulation of Wnt signaling^26^. To evaluate the effect of S-SCAM overexpression on these processes, we examined the total and synaptic β-catenin levels in male S-SCAM Tg mice. We found that both total (H) and synaptic (P2) amounts of β-catenin were not significantly changed (Fig. 6a–c). Therefore, the reduction in synaptic Axin1 amounts did not cause β-catenin stabilization in the Tg mice. These results suggest that synaptic Axin1 plays a role in GSK3β recruitment and S-SCAM overexpression does not directly affect the canonical Wnt signaling.

## Discussion

In this paper, we described the intriguing interplay of S-SCAM and Axins in the assembly of the GSK3β signaling complex at synapses. We also uncovered the role of Axins in the sex differences displayed in S-SCAM Tg mouse model of SCZ.

There are three main findings: First, we showed that Axin plays a pivotal role in the synaptic localization of GSK3β and the proper assembly of the GSK3β signaling complex involved in synaptic plasticity. S-SCAM inhibits these processes by binding to Axin in competition with GSK3β. Notably, Axin mediates the synaptic localization of GSK3β in a manner independent of the canonical Wnt signaling, which is reminiscent of Axin’s role in the axonal localization of GSK3β^25^. At this point, the molecular mechanism(s) responsible for the reduced Axin protein levels at synapses in S-SCAM overexpressing neurons remains unidentified. However, we suspect that increased glutamatergic activity at synapses might contribute to the reduction of synaptic Axin1 levels. S-SCAM Tg mice showed impaired synaptic GSK3β activity (shown by both reduced GSK3β protein levels and increased inhibitory phosphorylation), which is associated with increased CaMKII activity at synapses. Reduced GSK3β activity could lead to Axin reduction, since GSK3β stabilizes Axin via direct phosphorylation^36,37^ and dephosphorylated Axin is degraded^38,39^. Moreover, it is well known that increased glutamatergic activity promotes poly-ubiquitination of synaptic proteins^40,41^. Axin is a target for poly-ubiquitination and subsequent degradation by proteasomes^29^. Interestingly, under our experimental conditions, XAV939 did not increase Axin2 levels, unlike Axin1. In addition to preventing Axin degradation, XAV939 blocks the mRNA expression of Axin2 but not Axin1^23,42^. Therefore, it is plausible that Axin2 protein levels did not change by XAV939 because there is no new Axin2 protein synthesis to increase total Axin2 levels. Further studies await to verify this possibility.

Second, we demonstrated that GSK3β hypofunction is associated with the S-SCAM Tg mouse model. GSK3β is a key protein kinase strongly implicated in the pathogenesis of SCZ^43,44^. GSK3 hyperfunction is found in individuals with SCZ^45^. Consistently, genetic and molecular studies confirmed the key contribution of GSK3β hyperfunction in the pathogenesis of SCZ^44,46,47^. Paradoxically, hypofunction of GSK3β is found in patients with SCZ. Postmortem studies of SCZ patients revealed low GSK3β activity^48^, reduced protein levels^49,50^ (but see also^51^), and immunoreactivity^52^. Moreover, genetic association studies uncovered a single-nucleotide polymorphism that reduces GSK3β mRNA and protein levels in human subjects^53^. However, due to small sample sizes and high variability of the data, the pathophysiological significance of GSK3β hypofunction has remained unclear. To our knowledge, our studies provide the first preclinical evidence supporting the causal relationship of GSK3β hypofunction and the pathogenesis of SCZ.

Third, we identified mechanisms responsible for sex differences observed in S-SCAM Tg mice, which might be potentially relevant to SCZ. Sex differences are well documented in the humans with SCZ, which include disease risk, course, and outcome^15^. Men have 1.4-fold higher incidence of SCZ, display more severe symptoms and worse cognitive impairments, and are generally less responsive to antipsychotic treatments when compared to women^54^. Moreover, SCZ incidence in women greatly increases around menopause^55^. Based on sex differences of SCZ, the “estrogen hypothesis” was proposed in which the powerful female sex hormone estrogen plays a protective role against the development and severity of the disease. In clinical trials, supplemental estrogen treatment administered in conjunction with antipsychotics is beneficial for SCZ^55^. Our results suggest that Axin deficits at synapses are responsible for the male-specific GSK3β hypofunction in S-SCAM Tg mice. In female S-SCAM Tg mice, estrogen seems to buffer the Axin deficit at synapses by increasing Axin2 expression in neurons. Remarkably, Axin2 levels are increased to a level comparable to the elevated amounts of synaptic S-SCAM proteins (~ 1.5-fold)^14^. Therefore, the increase in Axin proteins seems sufficient to compensate for the increased S-SCAM levels and maintains GSK3β levels at synapses. These results are consistent with previous findings that, unlike Axin1, Axin2 shows an inducible expression pattern that is dependent on β-catenin/Tcf^34^. Moreover, it was shown that E2 activates β-catenin-dependent transcription in neurons^56^. Therefore, it is highly conceivable that E2 protects Axin levels at synapses in female S-SCAM Tg mice (and thereby GSK3β signaling complex) by promoting Axin2 transcription. Overall, our findings provide strong support for the estrogen hypothesis.

The S-SCAM Tg mice were generated by using the CaMKII promoter to drive the expression of S-SCAM transgene. Therefore, the Tg mice have elevated S-SCAM levels primarily in excitatory neurons of the forebrain area and most highly in the hippocampus^57^. Therefore, the sex differences observed in the Tg mice are likely caused by the excitation/inhibition imbalances in these principal neurons. Consistently, it is well documented that hippocampal functions are highly influenced by estrogen^58–60^. However, it remains to be determined whether sex differences of SCZ are also driven by the alterations of the glutamatergic function in the hippocampus and/or other brains regions.

We used cultured rat hippocampal neurons, which are reliable and amenable for molecular-genetic and pharmacological manipulations. These advantages allowed us to perform initial studies on the molecular mechanisms underlying sex-differences in S-SCAM Tg mice. We do not expect significant differences in rat vs mouse hippocampal neurons, since the major phenotypes of S-SCAM overexpression found from rat neurons were replicated in mouse neurons in vivo^1,14^. Further studies using the S-SCAM Tg mouse model will strengthen the role of Axin and E2 in sex-differences found in this study.

Finally, our studies provide a potential new therapeutic target for SCZ. Recent reports demonstratedthat chronic intraperitoneal XAV939 treatment of mice has an impact on the Wnt signaling in the brains, suggesting that XAV939 passes the blood–brain barrier and stabilizes Axin^61^. Therefore it would be interesting to investigate the effect of XAV939 on the SCZ-like behavioral deficits displayed in S-SCAM male Tg mice, especially on synaptic plasticity and working memory deficits.

## Methods

### Animals

S-SCAM Tg mice (C57BL/6J-Tg(Camk2a-Magi2)1Shlee/J; Jackson stock No: 027306) were maintained as described^14^. Timed pregnant Sprague-Dawley female rats were obtained from Envigo. All experimental procedures involving the mice and rats were performed in accordance with the relevant guidelines and regulations and approved by the Institutional Animal Care and Use Committee in the Medical College of Wisconsin. All methods are reported in accordance with ARRIVE guidelines.

### Cultured rat hippocampal neurons and transfection

Dissociated rat hippocampal neuron culture was prepared from E18 embryos (both sexes were used) of Sprague Dawley rats and maintained in Neurobasal medium supplemented with B27 and Pen/Strep (ThermoFisher Scientific) as described previously^62^. Hippocampal neurons were transfected at div14 using Lipofectamine 2000 as described previously^63^.

### Reagents

XAV939 and β-estradiol (E2) were purchased from Tocris. Actinomycin D was obtained from Sigma.

### Immunocytochemistry

Transfected neurons were fixed at 2 days post-transfection. Immunocytochemistry was performed as described^1,63^. Briefly, hippocampal neurons were first fixed in 4% formaldehyde/1 × PBS/4% sucrose for 15 min followed by membrane permeabilization in 0.5% TritonX-100/1 × PBS for 10 min. After washing three times in 1 × PBS for 10 min, fixed neurons were incubated with primary antibodies diluted in 1 × GDB (0.1% gelatin, 0.3% Triton X-100, 0.45 M NaCl, 17.7 mM sodium phosphate buffer, pH 7.4) in a humidified chamber overnight at 4 °C. Primary antibodies used and their dilution factors are: rabbit anti-GSK3β (1:100; Abcam), rat anti-HA (1:250; Roche), or mouse anti-myc antibodies (1:100; Santa Cruz Biotechnology). Bound primary antibodies were detected using Alexa 488-(Thermofisher) or Cy3-conjugated secondary antibodies (Jackson Immunoresearch Laboratories).

### Immunocytochemical image acquisition and analyses

Images were acquired by using a Nikon C1 plus laser scanning confocal microscope and 60 × objective (NA1.4). Acquired images (z-series stacks) were first converted to projection images (with maximal projection option) for analyses. Both image acquisition and analyses were done in a blind manner. To measure synaptic GSK3β intensities, 15–30 dendritic spine regions were randomly selected from the dendrites of transfected neurons based on GFP or myc fluorescence (overexpressed S-SCAM is highly enriched in dendritic spines^1^) using SynPAnal software^64^. After applying threshold, integrated intensity values from individual spines were obtained and average values of them were calculated per neuron basis in Excel. All data were transferred to GraphPad Prizm software for computation and graphical representation.

### Western blotting analyses

Total homogenate (H) and synaptosome (P2) fractions of mouse forebrain tissues were prepared as described before^27^. For the preparation of TCL from cultured neurons, pre-heated 2 × SDS sample buffer (65 °C) was directly added to the wells of culture dishes after washing once in ice-cold 1 × PBS. The H fractions, P2 fractions, or TCLs were separated on SDS–polyacrylamide gels and transferred onto PVDF membrane (Immobilon-P; Millipore). The membrane was blocked in 6% nonfat-dried milk/1 × TBS-T. Primary antibodies used in the studies are: mouse anti-GSK3β (1:1000; Cell Signaling Technology), rabbit anti-phospho-GSK3β (Ser9; 1:1000; Cell Signaling Technology), rabbit anti-GAPDH (1:1000; Cell Signaling Technology), rabbit anti-Axin1 (1:1000; Cell Signaling Technology), mouse anti-β-catenin (1:500; Millipore), mouse anti-CaMKIIa (1:100; ThermoFisher Scientific), mouse anti-CaMKIIb (1:1000; ThermoFisher Scientific), rabbit anti-phospho-CaMKII (Thr286; 1:1000; Phospho Solutions), rabbit anti-Akt (1:1000; Cell Signaling Technology), rabbit anti-phospho Akt (Thr308; 1:1000; Cell Signaling Technology), rabbit anti-Axin2 (1:1000; Abcam), and mouse anti-NSF (1:2000; Millipore). Primary antibodies were diluted in the blocking buffer and incubated overnight at 4 °C. After washing in 1 × TBS-T, membranes were further incubated for 1 h with HRP-conjugated secondary antibodies (GE Healthcare or Cytiva). Bound antibodies were detected by using SuperSignal West Pico Plus Chemiluminescent substrates (Thermo Scientific) and images were acquired by using luminescent image analyzer (ImageQuant LAS4000, GE Healthcare).

### Statistical analyses

All neuron experiments were performed at least in triplicate using independent batches of hippocampal neuron cultures. All data values represent means ± s.e.m. For multiple group comparisons, one-way ANOVA with Tukey’s multiple comparison post hoc test were performed using the GraphPad Prizm software. Welch’s t test (unpaired) was used to determine the statistical significance for two groups. *p* < 0.05 was considered significant.

## Supplementary Information

Supplementary Figures.

## Data Availability

All materials, data, and associated protocols will be promptly made available to readers without undue qualifications in material transfer agreements.
