# Supplementary material for: S-SCAM inhibits Axin-dependent synaptic function of GSK3β in a sex-dependent manner
Source: Sci Rep. 2022 Mar 8;12:4090. doi: 10.1038/s41598-022-08220-1 (PMC8904762; doi:10.1038/s41598-022-08220-1)
Supplement: Supplementary file 1 — Supplementary Figures. [file 41598_2022_8220_MOESM1_ESM.pdf]

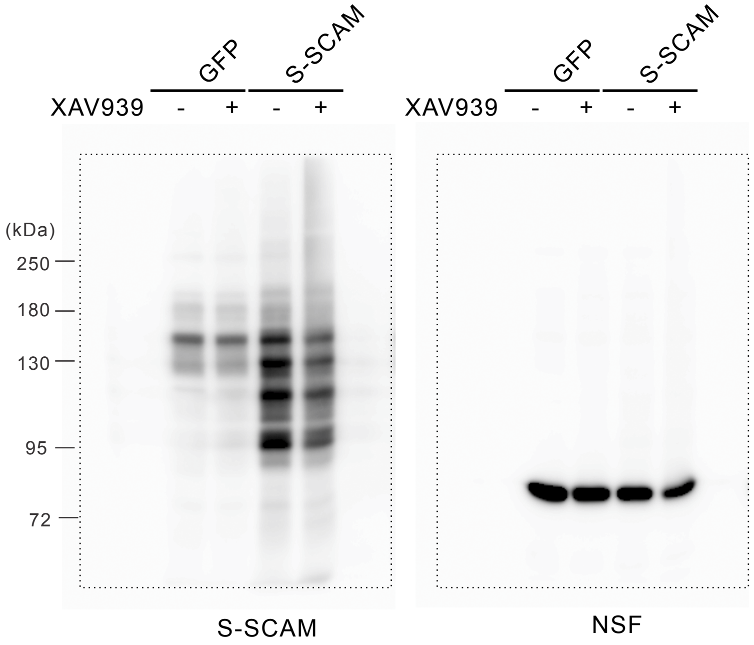

Supplementary Figure 1. Total S-SCAM protein levels in GFP- and S-SCAM-virus infected neurons. Related to Fig. 4a. NSF is used as loading control. Uncropped full-size blots are shown.

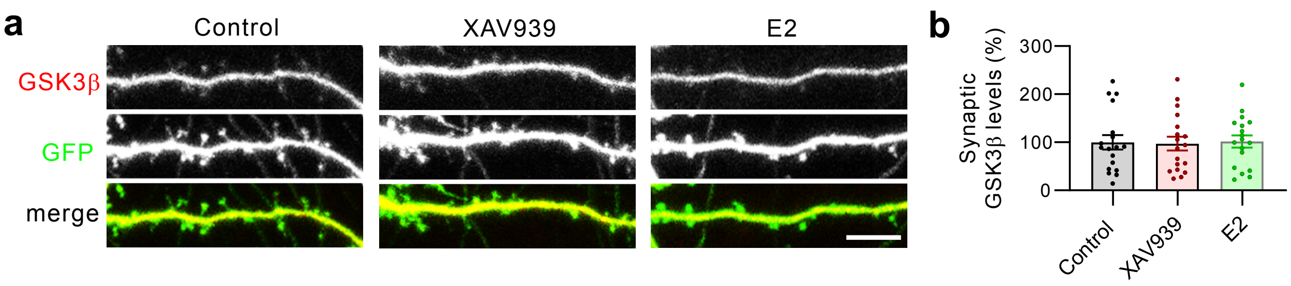

Supplementary Figure 2. Effect of XAV939 and E2 on synaptic GSK3β levels in EGFP-transfected control neurons. Related to Fig. 4d,e and Fig. 5h,i. (**a**) Representative images (**b**) Quantification of the data. n = 18 per condition.

Fig. 1a

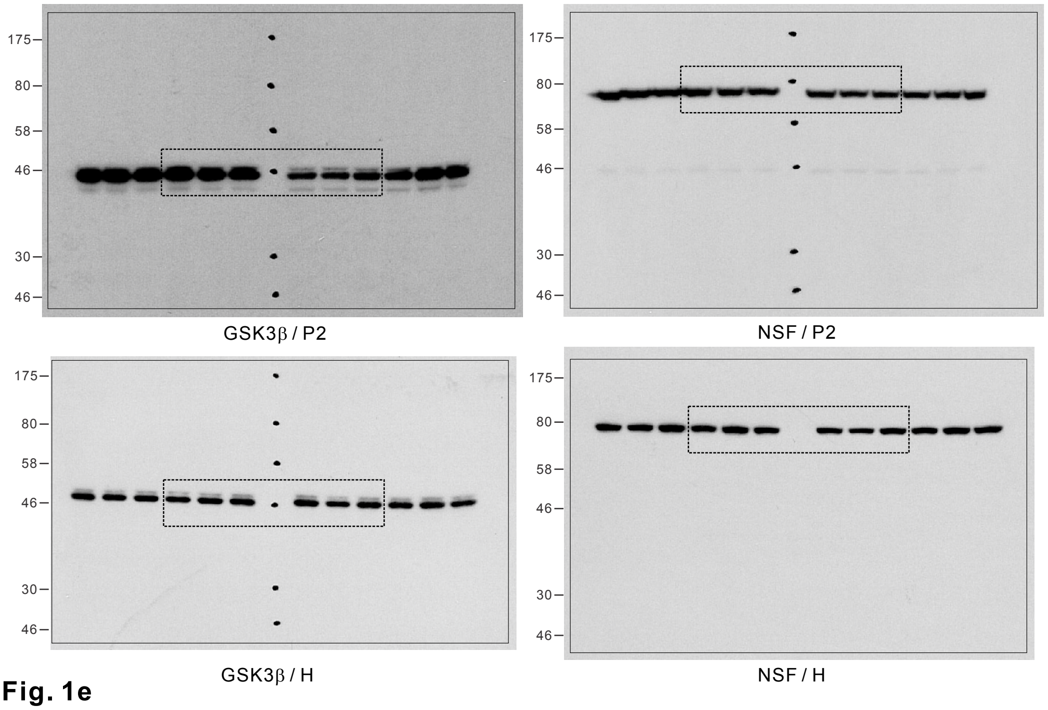

Fig. 1e

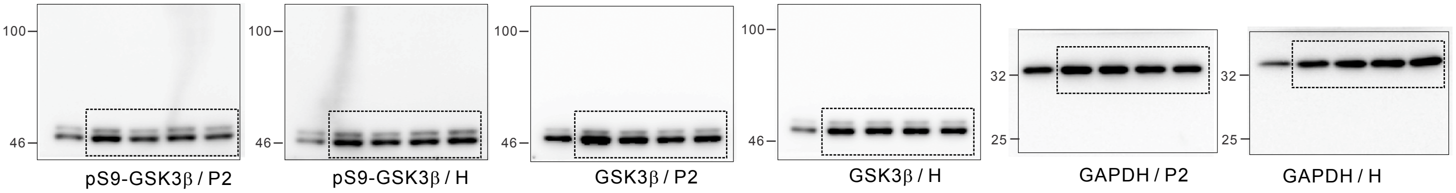

Fig. 2a

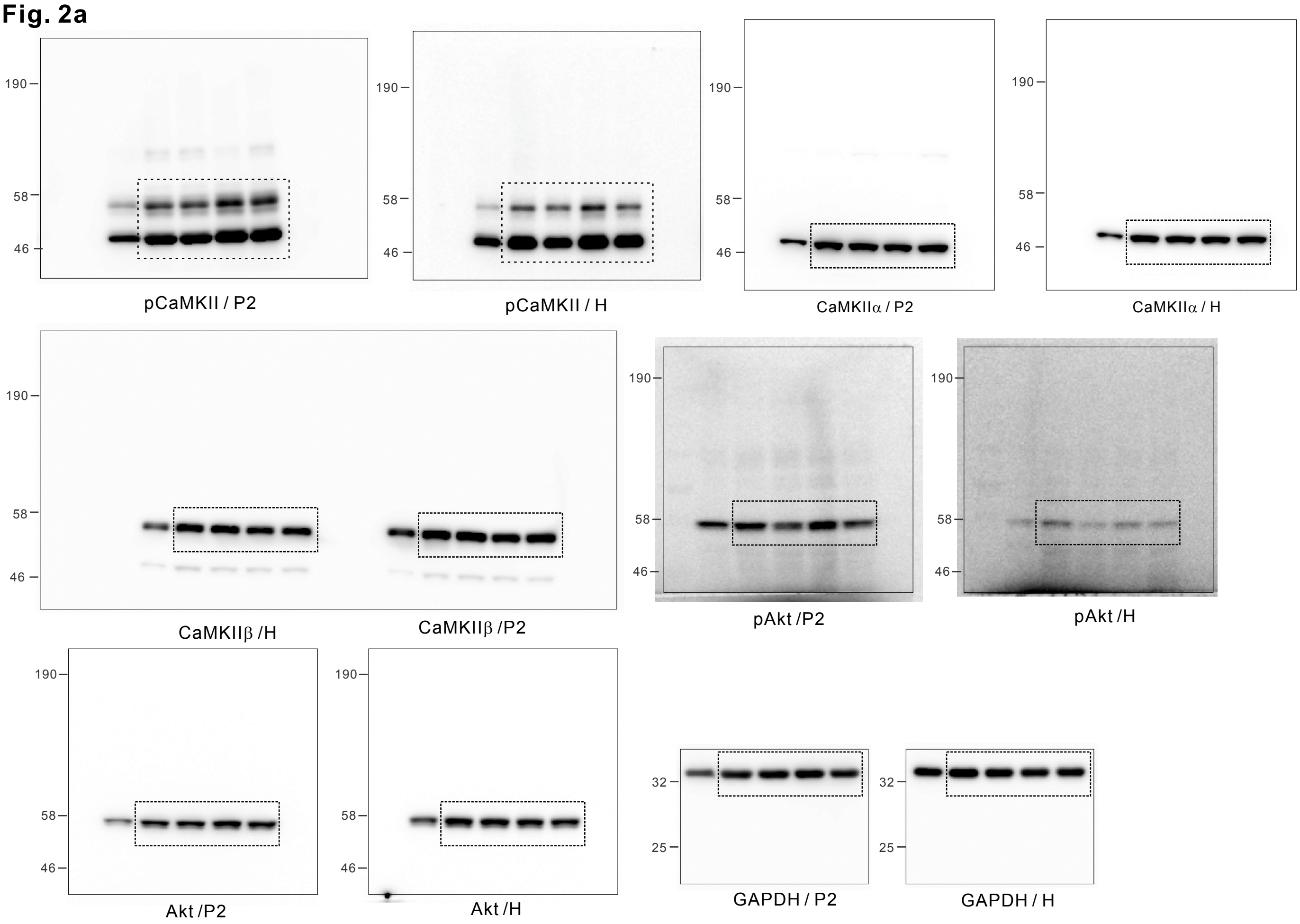

Supplementary Figure 3. Uncropped original images of blots. Membrane edges are outlined by solid black lines and cropped areas are indicated by dotted boxes. Positions of size markers are indicated on the left (in kDa). Note that blots were precut before the antibody incubation for probing with multiple antibodies.

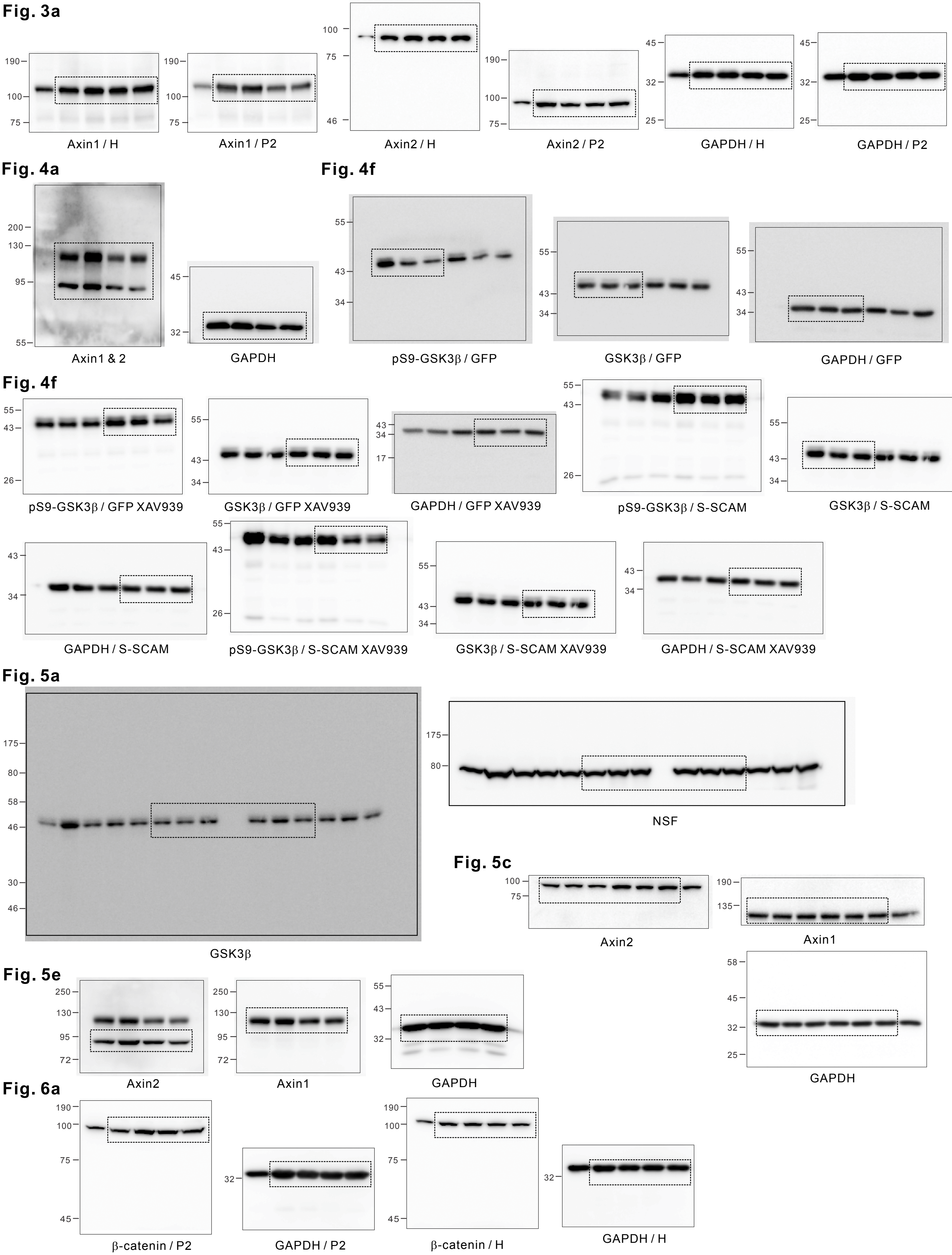

Supplementary Figure 3. Uncropped original images of blots. *Continued*.
